# Acute Sheehan’s syndrome manifesting initially with diabetes insipidus postpartum: a case report and systematic literature review

**DOI:** 10.1007/s00404-021-06294-2

**Published:** 2021-11-15

**Authors:** Gregor Leonhard Olmes, Erich-Franz Solomayer, Julia Caroline Radosa, Panagiotis Sklavounos, Philipp Agne, Stefan J. Schunk, Bashar Haj Hamoud

**Affiliations:** 1 Department of Gynecology, Obstetrics and Reproductive Medicine, Saarland University Hospital, Homburg, Germany; 2 Department of Internal Medicine IV – Nephrology, Saarland University Hospital, Homburg, Germany

**Keywords:** Sheehan’s syndrome, Diabetes insipidus, Peripartum hemorrhage, Case report, Prolactin

## Abstract

**Purpose:**

Acute Sheehan’s syndrome is a rare, but potentially life-threatening, obstetric event that can be complicated by diabetes insipidus. Little information on the diagnosis and treatment of Sheehan’s syndrome with diabetes insipidus is available. We report on a 28-year-old patient who developed acute Sheehan’s syndrome with diabetes insipidus after giving birth, and on a systematic review of similar cases.

**Methods:**

We performed a systematic review of the literature cataloged in PubMed and Google Scholar using the keywords “Sheehan syndrome” OR “Sheehan's syndrome” AND “diabetes insipidus” to identify relevant case reports published between 1990 and 2021. Eight Reports met the inclusion criteria (English-language abstracts available, onset in the puerperium, information about the day of the onset).

**Results:**

In the present case, postpartum curettage was necessary to remove the residual placenta. The total amount of blood loss was severe (2500 ml). On the second day postpartal, the patient developed polyuria. Laboratory analysis revealed hypernatremia with increased serum osmolality and decreased urinary osmolality. Hormone analysis showed partial hypopituitarism involving the thyroid, corticotropic, and gonadotropic axes. The prolactin level was elevated. Brain magnetic resonance imaging showed pituitary gland infarction. Desmopressin therapy was initiated and resolved the polyuria. Hormone replacement therapy was administered. Four months later, the patient was well, with partial diabetes insipidus. The literature review indicated that this case was typical in terms of symptoms and disease onset. Most reported cases involve hypotension and peripartum hemorrhage, but some patients without hemorrhage also develop Sheehan’s syndrome. Elevated prolactin levels are uncommon and associated with poor prognosis in patients with Sheehan’s syndrome.

**Conclusion:**

Acute Sheehan’s syndrome with diabetes insipidus involves nearly all pituitary hormone axes, indicating severe disease. Prolactin elevation could suggest that a case of Sheehan’s syndrome is severe.

## Introduction

Sheehan’s syndrome is characterized by ischemic necrosis of the anterior pituitary gland after severe postpartum hemorrhage, with hormonal insufficiencies ranging from single pituitary hormone insufficiency to total hypopituitarism [1]. The pituitary gland undergoes physiological enlargement during pregnancy, with a physiological increase in lactotroph cell mass and hypertrophy of its anterior portion, as determined by autopsies and magnetic resonance imaging (MRI) [2, 3]. Ischemia of the hypertrophied anterior pituitary gland during severe hemorrhage is thought to be the cause of Sheehan’s syndrome [4]. The onset of disease ranges from an acute onset in the puerperium to several years after birth [5–7]. The role of autoantibodies remains unclear for etiology [6].

Central diabetes insipidus is a rare complication of Sheehan’s syndrome [8–10]. Here, we present the case of a woman with Sheehan’s syndrome manifesting initially with central diabetes insipidus in the context of a systematic literature review.

## Case report

### Clinical presentation

A healthy 28-year-old primigravida gave birth to a male infant weighing 3030 g (12th percentile; Apgar score (1-min 9/5-min 10/10-min 10) at 40 gestational weeks in the Department of Gynecology, Obstetrics and Reproductive Medicine, Saarland University Hospital, Homburg, Germany, a tertiary center of perinatal medicine. The delivery occurred without complication with the infant in the left anterior occiput position after mediolateral episiotomy (umbilical artery pH 7.320, Base excess (BE) − 8.4; umbilical vein pH 7.329, BE − 7.6). Uterotonic medication was administered prophylactically, and the placenta was delivered 23 min after childbirth.

Thereafter, the patient entered shock with hypotension (arterial blood pressure 90/50 mmHg, heart rate 80 beats per minute) by an atonic bleeding (Table 1). Clinical examination and sonography revealed the presence of residual placenta in the uterine cavity. It was removed by emergency curettage performed under general anesthesia. During this procedure, the patient’s arterial blood pressure normalized, ranging from 125/80 to 100/50 mmHg. The urine production was 125 ml/h.

**Table 1:** Clinical parameter during shock

Arterial blood pressure	90/50 mmHg
Heart rate	80/min
Blood loss	2500 ml
Urine production	125 ml/h
Hemoglobin level preoperatively	11.8 g/dl
Hemoglobin level postoperatively	9.3 g/dl

To treat the atony, we followed the algorithm of the interdisciplinary D-A-CH consensus group PPH 2014 [11]. Oxytocin (Oxytocin 10 I.E. HEXAL^®^ 10 I.E./ml), sulprostone (Nalador-500^®^, 500 µg sulprostone Jenapharm^®^) and misoprostol (Cytotec^®^ 200 μg Misoprostol Tabs Pfizer^®^) were administered and a Bakri^®^ balloon catheter (Bakri Postpartum Ballon COOK^®^ MEDICAL) was placed into the uterine cavity for 24 h. The mediolateral episiotomy was repaired with sutures. Tranexamic acid (2 g, Cyklokapron^®^ Pfizer^®^ 1000 mg/10 ml), fibrinogen (2 g, Haemocomplettan^®^ P 1 g, CSL Behring^®^), and two erythrocyte concentrates (each 300 ml*)* were administered perioperatively. The bleeding stopped and the patient’s hemoglobin level decreased (to 9.3 g/dl from 11.8 g/dl preoperatively). The total volume of blood loss was 2500 ml (Table 1).

Postoperatively, the patient was monitored overnight in the department’s intermediate care unit. The balloon catheter was removed after 24 h, and the lochia was determined to be normal. The patient was transferred to the postpartal ward the following morning. In the evening of the first postpartal day, the patient reported hot flashes and headache. She could not breastfeed because of agalactorrhea (Table 2). In the morning of the second postpartal day, the patient reported polydipsia (Table 2). Her urinary production was 14 l/day (580 ml/h). The patient was transferred back to the intermediate care unit for monitoring. A central venous catheter was installed to start an infusion with electrolyte solutions 100 ml/hour (Sterofundin^®^, 1000 ml BRAUN^®^). Laboratory analysis on the second day after delivery revealed hypernatremia (sodium 148 mmol/l), hyperchloremia (chloride 116 mmol/l), serum hyperosmolality (305 mosmol/kg), and decreased urinary osmolality (66 mosmol/kg) (Table 2). The patient’s hemoglobin level decreased over the second and third postpartum days to 6.5 g/dl, and three erythrocyte concentrates (each 300 ml) were transfused on the third day after delivery (Table 2). After this transfusion, the patient’s hemoglobin level stabilized at 8.0 g/dl.

**Table 2:** Timeline of symptoms, diagnostic assessment and therapeutic intervention

Day after delivery	Symptoms	Diagnostic assessment	Therapeutic intervention
First day	Hot flashes, headache, agalactorrhea	–	–
Second day	Polydipsia, polyuria	Laboratory analysis of sodium, chloride, osmolality of serum and urine	Infusion with electrolyte solutions
Third day	Drop of hemoglobin level 6.5 g/dl,	Brain MRI scan, analysis of hormones	Transfusion of three erythrocyte concentrates
Fourth day	–	–	Desmopressin therapy and hormone replacement

### Diagnostic assessment

The patient was transferred to the hospital’s nephrology department for further diagnostic workup and treatment on the third day after delivery. Polyuria featuring hypernatremia, hyperchloremia, serum hyperosmolality, and decreased urinary osmolality led to the diagnosis of diabetes insipidus. The brain MRI examination performed on the third day after birth revealed mild ischemic infarction of the pituitary gland compatible with Sheehan’s syndrome with central diabetes insipidus. (Table 2). Hormonal assessment revealed partial hypopituitarism, including hypothyroidism and Addison’s disease (Table 3). Prepartal hormone values were not available. The patient’s thyroid-stimulating hormone level was normal (0.49 µIU/ml) and her free T3 and T4 levels were low (1.71 pg/nl and 0.63 µg/ml, respectively). Her cortisol level was in the low normal range (15.2 µg/dl), interpreted as reflecting a lack of adrenocorticotropic hormone.

**Table 3:** Hormone values postpartum and 6 weeks after birth

Hormone [units] (normal range)	Postpartum	6 weeks after birth
Thyroid-stimulating hormone [µIU/ml] (0.27–4.20)	0.49	0.5
Free T3 [pg/ml] (2.0–4.4)	1.7	2.5
Free T4 [ng/dl] (0.93–1.70)	0.63	1.3
Prolactin [µIU/ml] (102–496)	1033	300
Cortisol [µg/dl] (6.2–19.4)	15.2	31.7
Luteinizing hormone [mIU/ml] (premenopausal 1.0–12.6, postmenopausal 7.7–58.5)	< 0.1	3.8
Follicle-stimulating hormone [mIU/ml] (premenopausal 1.7–21.5, postmenopausal 25.8–134.8)	< 0.1	10.1
Estradiol [pg/ml] (premenopausal 12.4–398, postmenopausal < 5–138)	15	42.2

Additional laboratory examinations, including the analysis of sex hormone and gonadotropin levels, were performed in the hospital’s endocrinology department on the fourth day after birth. The levels of luteinizing hormone (LH; < 0.1 IU/ml) and follicle-stimulating hormone (FSH; < 0.1 mIU/ml) were decreased, confirming the patient’s postmenopausal status (Table 3). The levels of sex hormones, such as estradiol (15 pg/ml) and progesterone (0.238 ng/ml), were low. Additional analysis revealed that the anti-Müllerian hormone level was also low (0.67 ng/ml). The prolactin level was high (1033 µlU/ml), but the patient remained unable to breastfeed.

### Therapeutic intervention

Daily administration of desmopressin (Nocutil^®^ 0,1 mg/ml-nasal spray, AGOPHEA^®^) starting on the fourth day after delivery as a nasal spray led to a normalized urine production, and thus to a normalized urine and serum osmolality (both 106 mosmol/kg). Under the daily treatment, the patient got transient hyponatremia (sodium 122 mmol/l), that resolved after a 3-day pause in the desmopressin treatment (sodium 141 mmol/l). Thereafter, the treatment was applied depending on polyuria and nycturia. The patient’s hypothyroidism was treated with l-thyroxine (50 µg/day, l-thyroxine Henning^®^, Sanofi^®^) and her Addison’s disease was treated with hydrocortisone (20 mg in the morning, 10 mg at noon, hydrocortisone GALEN^®^ 10 mg Tabs). A gynecological endocrinologist initiated sequential hormone replacement therapy for the patient with the transdermal application of estradiol gel (Gynokadin^®^ estradiol 0.6 mg/g, twice a day) and additive cyclic oral progesterone (Utrogest^®^ 100 mg progesterone DR. KADE BESINS^®^, two tabs daily) for 12 days every two weeks. This specialist recommended FSH/LH application via hormone pump or pen for future pregnancies. Thirteen days after delivery, the patient was discharged in stable condition.

### Follow-up

The patient underwent regular, in the first week after discharge daily, follow-up examinations in the hospital’s ambulatory unit. Six weeks after delivery, her hormone levels had normalized (Table 3). Four months postpartal, the patient felt well and had normal urine production, but also partial diabetes insipidus. This condition was treated with desmopressin nasal spray (twice per day). Her serum sodium concentration and serum and urinary osmolality were normal (138 mmol/l, 294 mosmol/kg, and 820 mosmol/kg, respectively).

## Discussion

Sheehan’s syndrome is a rare complication of pituitary disorders that occurs during pregnancy separate from lymphocytic hypophysitis and silent pituitary adenoma [12, 13]. It is seldom encountered in developed countries [12]. The prevalence of Sheehan’s syndrome amounted from 5.1 per 100 000 women in Iceland in 2009. No patient of these collective showed signs for the involvement of the posterior pituitary gland [14]. Diabetes insipidus in patients with Sheehan’s Syndrome occurs in 5% [13, 15, 16]. For patients with acute Sheehan’s Syndrome, an electronic literature search on PubMed and Google Scholar by Matsuzaki et al. could identify 21 cases of patients with Sheehan’s Syndrome. Four of these cases presented a diabetes insipidus accompanied by Sheehan’s Syndrome [7, 8, 13, 17, 18].

Sheehan and Whitehead reported that most cases of Sheehan's syndrome manifest with anatomical lesions in the posterior pituitary gland and hypothalamic secretory neurons [19]. The impairment of antidiuretic hormone secretion, which causes diabetes insipidus, occurs only when a large portion of the neurohypophysis is destroyed [20, 21]. The degree of polyuria depends on the proportion of the anterior pituitary gland that is functional [21]. Bakiri et al. reported decreased urinary concentration ability in patients with slow-onset postpartum hypopituitarism. They underwent a dehydration test followed by the administration of desmopressin, reflecting a high prevalence of partial diabetes insipidus among patients with Sheehan’s syndrome [22]. Isolated diabetes insipidus is described after postpartum hemorrhage [23].

Diabetes insipidus apart from Sheehan’s syndrome is rare during pregnancy and is caused by the increased placental production of vasopressinase, which inactivates circulating vasopressin [24]. This condition is usually transient and disappears a few days after delivery [24].

We performed a systematic review of the literature cataloged in PubMed and Google Scholar using the keywords “Sheehan syndrome” OR “Sheehan's syndrome” AND “diabetes insipidus” to identify relevant case reports published between 1990 and 2021. The search yielded 32 PubMed entries and 838 Google Scholar entries. From these, we selected articles with at least English-language abstracts available that described cases of acute Sheehan syndrome with onset in the puerperium in combination with diabetes insipidus. Information about the day of the onset was mandatory.

Eight reports were included in the analysis, each of them describing one patient [8, 10, 13, 17, 18, 25–27].

The following data were extracted from the reports: patient age; gravida/para status; mode of delivery; amount of blood loss described as normal or severe; cause of hemorrhage, hypotension, or shock; interval from birth to symptom onset; presence of polyuria; presence of symptoms including headache, fatigue, and agalactorrhea; other hormone disorders (e.g., hypothyroidism, adrenal insufficiency, hypogonadism, hypoprolactinemia, somatotropic axis failure); prolactin level; performance of brain MRI or computed tomography (CT) examination; treatment of diabetes insipidus; and follow-up assessment/treatment.

The characteristics of the cases are summarized in Table 4. Eight patients were included in the review. Patient age was provided in seven case reports [8, 10, 13, 17, 18, 25, 27]. The median patient age was 34.8 (range, 24–45) years, thus older than our patient. Our case is related to a primigravida with vaginal delivery. Five reports described the patents’ gravida/para status; the latter ranged from primipara to multipara [8, 10, 17, 25, 27]. Twins were delivered in two cases [10, 17]. The delivery mode was reported for seven cases; four patients delivered by caesarian section and three had vaginal deliveries [8, 10, 13, 17, 18, 25, 27].

**Table 4:** Characteristics of cases identified by systematic literature review

Authors	Olmes et al.	Rahmani Tzvi-Ran et al. [25]	Robalo et al. [10]	Kumar et al. [8]	Catinois et al. [26]	Wang et al. [18]	Kan and Calligerous [17]	Dejager et al. [13]	Kuhn et al. [27]
Year of publication	2021	2019	2012	2011	2004	2002	1998	1998	1998
Patient age (years)	28	24	45	36	N/A	32	32	32	43
Gravida/para status	1/1	2/2	2/1	Multipara	N/A	N/A	Multipara	N/A	Multipara
Mode of delivery	Vaginal	Emergent caesarean section	Vaginal (twins, preterm)	Vaginal	N/A	Cesarean section	Cesarean section (twins)	Vaginal	Cesarean section
Blood loss	Severe	Normal	Severe	Severe	Severe	Severe	Severe	Normal	Severe
Cause of hemorrhage	Retained placental fragments	N/A	Retained placental fragments	Atonic bleeding	N/A	Persistent bleeding from uterus	N/A	N/A	Amniotic fluid syndrome
Hypotension or shock	Yes	No	Yes	Yes	Yes	Yes	N/A	Yes (during epidural anesthesia)	Yes
Interval from birth to symptom onset	24 h	11 days	15 days	4 days	2 days	19 days	24 h	3 days	15 days
Polyuria	Yes	Yes	Yes	Yes	Yes	Yes	Yes	Yes	N/A
Symptoms									
Headache	Yes	Yes	Yes	N/A	Yes	N/A	N/A	Yes	N/A
Fatigue	N/A	Yes	N/A	N/A	N/A	N/A	N/A	Yes	N/A
Agalactorrhea	Yes	Yes	Yes	N/A	Yes	N/A	N/A	N/A	N/A
Other	Hot flashes, polydipsia	Fatigue	Photophobia, thirst	N/A	Amenorrhea	N/A	N/A	N/A	N/A
Accompanying hormone disorders									
Hypothyroidism	Yes	Yes	Yes	N/A	N/A	Yes	N/A	No	Yes
Adrenal insufficiency	Yes	Yes	Yes	N/A	N/A	Yes	N/A	Yes	Yes
Hypogonadism	Yes	Yes	Yes	N/A	N/A	Yes	N/A	Yes	Yes
Hypoprolactinemia	No	Yes	Yes	N/A	N/A	No	N/A	Yes	Yes
Somatotropic axis failure	N/A	N/A	N/A	N/A	N/A	N/A	N/A	Yes	Yes
Prolactin level (µIU/ml; normal range 102–496)	Elevated (1033)	Low (148)	Low (302)	Low	N/A	Elevated	N/A	45.5	N/A
Brain MRI or CT	Yes	Yes	Yes	N/A	Yes	N/A	N/A	Yes	Yes
Diabetes insipidus treatment	Desmopressin	Desmopressin	Desmopressin	Desmopressin	N/A	Desmopressin	Desmopressin	Desmopressin	N/A
Follow-up (time after birth)	Well-being, desmopressin	Well-being, desmopressin (10 months)	Hormone replacement (12 months)	Desmopressin, hormone replacement (12 months)	N/A	N/A	N/A	Desmopressin (24 months)	N/A

The estimated blood loss was reported for all cases, and ranged from normal (two cases) [13, 25] to severe hemorrhage (six cases) [8, 10, 17, 18, 25, 27]. The term normal is defined as blood loss due 500 ml for a vaginal birth and due 1000 ml for caesarean section according to German guidelines [28]. A hemorrhage of 500–1500 ml is usually tolerated without symptoms of shock or hypotension [29, 30]. In line with the majority of reported cases, our case presented a severe hemorrhage with an estimated blood loss of 2500 ml. The hemorrhage required a transfusion immediately perioperatively and three days postpartal. Sheehan’s Syndrome is usually seen after severe peripartal hemorrhage with shock and hypotension [1, 31].

Four reports described the reasons for hemorrhage, which included the retention of placental fragments and/or atonic bleeding (as in our case), persistent bleeding from the uterus, and amniotic fluid syndrome [8, 10, 18, 27].

Six patients experienced hypotension or shock [8, 10, 13, 18, 26, 27] as in our case too and one patient had no hypotension [25]; no information on this factor was available for the remaining patient [17]. One patient had hypotension during epidural anesthesia, which has been described as a cause of Sheehan’s syndrome [13].

The interval between birth and the initial manifestation of symptoms, reported in all cases, ranged from 24 h to 19 days after delivery [8, 10, 13, 17, 18, 25–27]. The median was 8, 8 days. Our patient developed symptoms during the first 24 h.

Seven patients had polyuria [8, 10, 13, 17, 18, 25, 26]; no information on this factor was provided for the remaining patient [27]. Other symptoms were reported for four cases; they included headache (*n* = 4), fatigue (*n* = 2), agalactorrhea (*n* = 3), photophobia (*n* = 1), thirst (*n* = 1), and amenorrhea (*n* = 1) [10, 13, 25, 26]. Headache has been described as a main symptom of sellar mass apoplexy in patients with Sheehan’s syndrome [13]. In line with the symptoms mentioned above, our patient complained about headache, agalactorrhea and hot flushes, followed by polyuria and polydipsia on the day after.

Accompanying hormone disorders were described in five cases [10, 13, 18, 25, 27]. They were hypothyroidism (*n* = 3), adrenal insufficiency (*n* = 4), hypogonadism (*n* = 4), hypoprolactinemia (*n* = 3), somatotropic axis failure (*n* = 1), and panhypopituitarism (*n* = 1). Of these, our case demonstrated hypothyroidism, adrenal insufficiency and hypogonadism and in contrast hyperprolactinemia.

Patients’ prolactin levels were reported for five cases; they were low in four cases [8, 10, 13, 25] and elevated in one case, in which the patient had severe peripartum hemorrhage leading to chronic kidney disease [18]. Prolactin levels are typically low in patients with Sheehan’s syndrome [32]; the elevated levels found in the single previously reported case and in our case are atypical and may be associated with the severe form of this syndrome. Kelestimur reports on a patient with Sheehan’s syndrome and hyperprolactinemia, in which the hyperprolactinemia disappeared after the reduction of thyroxine to a suboptimal level [33]. A case report describing a patient with pituitary metastasis indicates that prolactin levels can be elevated during panhypopituitarism with a poor prognosis [34]. Oxytocin is also known to lead to prolactin secretion, which could explain the elevated prolactin level in our patient, although oxytocin was likely administered in the other cases included in this review [35]. Prolactin may be involved in lactotroph cell recovery in patients with Sheehan’s syndrome; one such patient was reported to lactate during a second pregnancy [36].

In five cases included in this review, diagnostic assessment included brain MRI or CT examination [10, 13, 25–27]. Similar to these cases, an MRI scan of the brain was performed in our patient too, to confirm the infarct of the pituitary gland. For patients with diabetes insipidus, MRI examination is recommended to define differential diagnoses such as adenoma and infiltrative or inflammatory changes in the posterior pituitary gland [37].

Six reports mentioned the use of desmopressin to treat diabetes insipidus in the context of Sheehan’s syndrome [8, 10, 13, 17, 18, 25]. In this condition, it is a form of central diabetes insipidus [9]. We treated our patient with desmopressin too. Desmopressin, which treats polyuria and polydipsia, is the standard therapy for central diabetes insipidus during pregnancy and postpartum [24]. It is dosed empirically. Regular clinical control of symptoms and laboratory examination of serum and urinary osmolality are necessary. Desmopressin treatment is usually lifelong because diabetes insipidus is a permanent condition in most patients [37].

Follow-up assessment was documented for four of the cases included in this review. In one case, the patient is mentioned with well-being; all follow-up patients still required desmopressin treatment at 10, 12, and 24 months, respectively, after birth [8, 10, 13, 25]. Follow-up visits for our patient were arranged in the ambulatory every six weeks and they are ongoing.

### Conclusion

This article summarizes relevant cases of common literature, giving insight to clinical features of patients with Sheehan’s Syndrome and diabetes insipidus, which is part of severe forms of Sheehan’s syndrome. Most patients of the reviewed literature with diabetes insipidus and acute Sheehan’s syndrome show involvement of nearly all pituitary hormone axes after peripartal hemorrhage. An elevated prolactin level may provide a hint that a case of Sheehan’s syndrome is severe, as it may indicate major damage with the loss of pituitary mass.

## Data Availability

The dataset used and analyzed during the current study is available from the corresponding author on reasonable request.
